# Contemporary Theory and Research

**Published:** 1893-10-21

**Authors:** 


### Contemporary Theory and Research.

### BACTERIA UNDERGROUND.

Some investigations carried out by Dr. Houston, of Edinburgh, respecting the number of bacteria in the soil at different depths from the surface go to prove that the micro-organisms become less and less abundant as the depth from the surface increases. For example, the average number of germs in a gramme of soil examined which was taken from the surface was 1,687,799; at a depth of three feet this average fell to 173,807; and at a depth of six feet it was only 410. The deduction is that at a certain definite distance from the surface the soil would be sterile.

### SANSCRIT MEDICINE.

Mr. Stephenson, of Bombay, contributes to the *Pharmaceutical Journal* a curious account of a very ancient Sanscrit manuscript treating of medicine and containing prescriptions largely used by the hakims or native doctors in India. The favourite form of medication appears to be oil, and the process of distillation is a very primitive one. "A quantity of the bruised drug is mixed with a proportion of milk; this is left to macerate for four or five days, after which it is put into a vessel made of metal or glass. This vessel, which consists of two flask-shaped portions, the necks of which fit into one another, is now closed and the lower or empty part buried in the ground, whilst the upper part, which contains the drug, remains exposed above the earth. A fire is now kindled round the upper part of the vessel and the oil eventually collects below." As a specimen of these prescriptions may be noted the "oil of sulphur" warranted to cure every disease known, if taken internally in doses of one drop on betel leaf and prepared as follows: Take of purified sulphur six parts; juice of calves' dung a sufficiency. Rub the sulphur in a mortar with sufficient juice to wet it daily for three days, then distil. Some of the external remedies are very peculiar, and for swelling of the neck, from which many of the natives suffer, several curious applications are suggested. Mango seeds and horse-hoof parings burnt together in a pot and mixed with butter is one, while another consists of camel's bones and buffalo's horns in powder mixed with sweet oil in which the flowers of Camra indica have been boiled. But the favourite specific for the complaint is a necklace of black serpents' bones, strung together, and worn round the neck. This is, however, a costly prescription, since such necklaces are valued as high as eighty rupees. Mr. Stephenson thinks that "these remedies act very often simply as a mask or blind, while the patient is being subjected to rigorous hygienic treatment, otherwise it would be difficult to account for the many wonderful and authentic cures wrought by the native medicine men of this and similar countries."

### DISSEMINATION OF CHOLERA BY FLIES.

Dr. Simmonds, of Hamburg, has been making experiments which tend to show that flies play an important part in the spread of cholera. In this first experiment he captured nine flies from the room in which he had made the autopsy of a cholera patient. He enclosed them for forty-five minutes in a large flask where they could fly about at their ease, and in which the air in motion could dry up and neutralise the particles of virus adhering to them. He then introduced each one to a tube containing liquid gelatine, and at the end of two days the "plaques" were covered with colonies of baccillus virgulus. The second experiment produced precisely the same results, and Dr. Simmonds deduces that in cholera times too much care cannot be taken to prevent flies from gaining access to the sick room or dissecting chamber since they may carry the germs they have gathered to articles of food which will prove excellent "bouillons" for their culture.

